# One-Pot Synthesis of Bright Blue Luminescent N-Doped GQDs: Optical Properties and Cell Imaging

**DOI:** 10.3390/nano11112798

**Published:** 2021-10-22

**Authors:** Huaidong Wang, Chong Qi, Ailing Yang, Xiaoxu Wang, Jie Xu

**Affiliations:** 1 College of Physics & Optoelectronic Engineering, Ocean University of China, Qingdao 266100, China; wanghuai-dong@stu.ouc.edu.cn (H.W.); qichong@stu.ouc.edu.cn (C.Q.); 2 College of Food Science & Engineering, Ocean University of China, Qingdao 266003, China; wangxx0416@163.com (X.W.); xujie9@ouc.edu.cn (J.X.)

**Keywords:** ultrasonic-assisted hydrothermal, nitrogen doped graphene quantum dots, optical properties, photo-stability, cell imaging

## Abstract

High fluorescent graphene quantum dots (GQDs) are promising in bioimaging and optoelectronics. In this paper, bright blue fluorescent N-doped GQDs were synthesized using a ultrasonic-assisted hydrothermal method. The morphology, structure, surface chemistry, optical properties, and stability subject to photo-bleaching, temperature, pH and preservation period for the N-GQDs were investigated in detail using various microscopy and spectroscopy techniques. The results showed that the N-GQDs possessed an average size of 2.65 nm, 3.57% N doping, and up to 54% quantum yield (QY). The photoluminescence (PL) spectra of the N-GQDs are excitation dependent when excited in the range of 300–370 nm and excitation independent in the range of 380–500 nm for the core and surface states emission. The N-GQDs showed excellent photo-bleaching resistance and superior photo-stability. At room temperature and in the pH range of 3–8, the fluorescence of the N-GQDs was almost invariable. The N-GQDs can be stably preserved for at least 40 days. The average decay lifetime of the N-GQDs was 2.653 ns, and the radiative and nonradiative decay rate constants were calculated to be 2.04 × 10^8^ s^−1^ and 1.73 × 10^8^ s^−1^, respectively. The PL mechanism was qualitatively explained. The N-GQDs was used for cell imaging, and it showed good results, implying great potential applications for bioimaging or biomarking.

## 1. Introduction

Graphene (Gr) and its derivative graphene oxide (GO) have been a hot research direction in materials science in recent years [1,2,3,4,5,6,7]; however, applications of Gr in the fields of bioimaging and optoelectronics have been limited, because Gr is a zero-band-gap semiconductor and a non-fluorescent substance. Graphene quantum dots (GQDs) are small sheets of Gr with lateral size of less than 10 nm, with oxygen-containing groups at the edges. As a result of the quantum confinement effect and the lateral effect, GQDs are fluorescent matter. In addition to this, GQDs possess other outstanding advantages, such as their being green, nontoxic, chemically inert, possessing good aqueous solubility and excellent biocompatibility, and lending themselves to easy modification, rendering GQDs as one of the most promising fluorescent nanomaterials, superior to conventional fluorescent organic dyes and luminescent inorganic quantum dots, with extensive potential applications in bio-imaging [8,9,10], as drug carriers [11], and in disease diagnosis [12], optical sensors [13,14,15], solar cells [16,17,18], light emitting diodes [19], and photocatalysts [20]. Several methods have been developed for the fabrication of GQDs. Generally, these approaches can be classified into two types: top-down and bottom-up. Top-down methods are mainly based on cutting the large carbon materials into nanoparticles (NPs), such as through the chemical exfoliation of graphite NPs [21] and the hydrothermal cutting of oxidized graphene sheets [22]. Top-down methods may produce some toxic products [23] that are difficult to completely remove in post-treatment procedures, thus leading to environmental pollution, causing health hazards to humans, and also limiting their widespread use. Bottom-up approaches use small molecules containing C, H, and O as precursors, via solid phase pyrolysis or hydrothermal condensation, to obtain GQDs [24,25].

Nitrogen-doped GQDs (N-GQDs) may improve the fluorescent quantum yield (QY) by adjusting the photoluminescence (PL) range. This enhances biocompatibility, and therefore, the properties of N-GQDs have attracted a significant amount of research interest [26]. The direct pyrolysis of small molecules in solid phase [27,28,29] and via the hydrothermal route [12,30,31] has been used to prepare N-GQDs. The direct pyrolysis of small molecules in solid phase to obtain N-GQDs is simple and quick, but small molecules are easily over-carbonized, and large particles can readily be produced, leading to a product with a wide size distribution and complex post-treatment procedures. The negative effects the direct pyrolytic method described above restrict its widespread use in the preparation of N-GQDs. The hydrothermal route seems to be the key to solving the problems inherent in the direct pyrolysis of small molecules in solid phase [32]. The ultrasonic hydrothermal method has distinct advantages with respect to maintaining homogeneous reaction conditions to prevent the N-GQDs from agglomerating, as well as providing a shorter reaction time, milder reaction conditions, lower energy consumption, better stability, and good reproducibility. Thus, in this paper, an ultrasonic hydrothermal method was used to synthesize N-GQDs in one step, with citric acid (CA) as a precursor and L-glutamic (L-Glu) for N doping and as a passivant. Compared to the two-step approach (first fabrication of GQDs, then amino-modification), the one-step strategy is easier and saves time; more importantly, the L-Glu functionalized GQDs have the benefit of decreasing the surface defects of GQDs, thus enhancing the QY and stability, and improving biocompatibility. The as-prepared N-GQDs were characterized in detail and used for cell imaging. The results indicated that the N-GQDs possessed excellent photo-physical properties and were effective in BV2 cell imaging. In this regard, this new strategy overcomes the limitations of existing N-GQDs, by reducing the surface defects, increasing the QY and stability, and improving biocompatibility.

## 2. Experimental Section

### 2.1. Materials and Characterization

L-Glu, CA and Rhodamine B reagents were all of analytical grade and were purchased from Aladdin Reagent Co., Ltd. (Shanghai, China). Ultrapure water (18.2 MΩ·cm) was used as a solvent. Propidium iodide (95%), Dulbecco’s modified Eagle medium (DMEM) and fetal bovine serum (FBS) were bought from Gibco (Gaithersburg, MD, USA). Penicillin and streptomycin sulfate were purchased from Invitrogen.

An YZUR-100 (Shanghai Yan Zheng, Shanghai, China) ultrasonic hydrothermal reactor (220 V) was used to synthesize N-GQDs; the ultrasonic power could be adjusted within 250 W, and the stirring speed was 200 rad/min; the pressure in the cavity was controlled between 0.84–0.88 MPa. The ultraviolet-visible (UV-Vis) absorption spectra were determined using a UH5300 spectrophotometer (Hitachi, Japan) at room temperature. PL spectra were measured using a FluoraMAX-4 fluorescent spectrometer (Horiba JY, Edison, NJ, USA) with excitation/emission slit width of 2/3 nm. The crystalline structure of N-GQD solid powder was characterized using an X-ray diffractometer (XRD, Bruker D8 ADVANCE, BRUKER AXS, Germany) with Cu-Kα radiation (λ = 1.5406 Å). A high-resolution transmission electron microscope (HRTEM, Tecnai G2 F30 Hillsboro, OR, USA) was used to observe the morphology and lattice of the N-GQDs prepared on an ultra-thin carbon film. The size distribution of the N-GQDs was analyzed with Image J software. By coating the diluted N-GQDs on freshly dissociated mica, the surface morphology and thickness of N-GQDs were characterized using an atomic force microscope (AFM, Seiko-SPA400, Tokyo, Japan) with ScanAsys atomic imaging optimization technology. Fourier transform infrared (FTIR) spectroscopy was employed using a Nicolet iN10 FTIR spectrometer (Thermo Fisher Scientific, Waltham, MA, USA) with a resolution of 4 cm^−1^ from 4000 to 500 cm^−1^. The test sample was prepared by grinding, mixing then pressing of 2 mg powder of N-GQDs and 100 mg KBr powder. X-ray Photoelectron Spectroscopy (XPS) with a multifunctional imaging electron spectrometer (Thermo ESCALAB 250XI, America, with a radiation source Al K*α*-1486.6 eV) was used to analyze the elemental compositions and chemical bonds of the obtained N-GQDs. The fluorescence lifetime of the N-GQDs was measured using the time-correlated single photon counting (TCSPC) technique, an Edinburgh F900 time-resolved fluorescence spectrometer (FLS-980, Edinburgh, UK) with an LED excitation source (370 nm), and an electrically cooled red sensitive R928P photon-counting photomultiplier tube detector to obtain the fluorescence lifetime, with a monitor emission wavelength of 440 nm. An incubator (Thermo Fisher Scientific, Waltham, MA, USA) was employed to culture BV2 cells. Cell imaging was performed using a confocal laser scanning microscope (LSM, Nikon A1R HD25, Tokyo, Japan) with three semiconductor lasers (405, 488, and 532 nm).

### 2.2. Preparation of N-GQDs

**Scheme 1:**
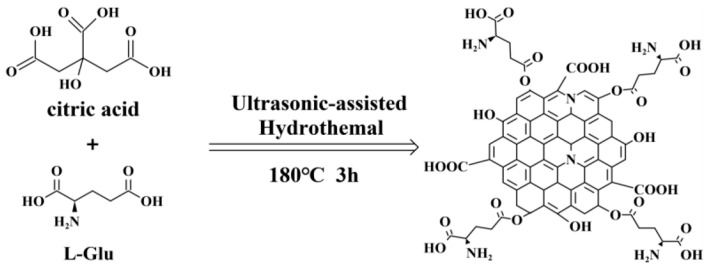
Schematic diagram of the formation of N-GQDs by ultrasonic-assisted hydrothermal method.

The ultrasonic-assisted method was used to fabricate the N-GQDs as shown in Scheme 1. The natural organic acid CA provided the carbon source, and the natural amino acid L-Glu supplied the amino group and part of the carbon source to achieve nitrogen doping. In the fabrication process, the reaction temperature (180 °C) was higher than the boiling point of CA (160 °C), but lower than that of the L-Glu (225 °C); therefore, CA was pyrolyzed, dehydrated and condensed to form GQDs. GQDs possess COOH and OH at the surfaces and edges; through the dehydration reaction, L-GLu was linked to GQDs, so the GQDs were functionalized by L-Glu in a single step, which is of great benefit for decreasing the surface defects on GQDs, thus enhancing the QY [33,34]. The amino groups in GQDs make them useful in the field of biomedicine [35]. The synthesis was optimized step by step (the experimental process is shown in the Supporting Information, see Figure S1 and Table S1). Typically, 3.6 g CA and 1.8 g L-Glu were dispersed into 30 mL deionized water; the solution was heated up to 180 °C in the ultrasonic hydrothermal reactor and kept at a constant temperature for 3 h; then the solution was cooled down to room temperature. By filtrating three times (filter with 0.22 μm hole) and dialysis (1000 D) for 18 h, a pure N-GQD solution was obtained. After freeze drying, solid-phase N-GQDs were obtained and stored in a refrigerator at 4 °C for later use.

## 3. Results and Discussion

### 3.1. Morphology and Structure of the N-GQDs

**Figure 1:**
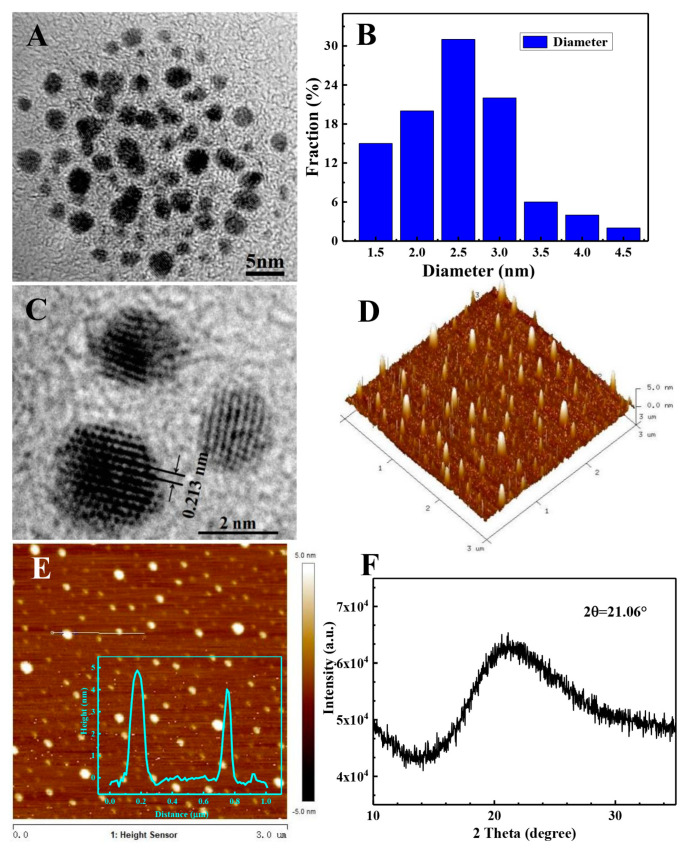
(**A**) TEM, (**B**) size distribution, (**C**) HRTEM, (**D**,**E**) AFM, and (**F**) XRD pattern of the N-GQDs.

The morphology and structure of the N-GQDs were characterized in detail using TEM, HRTEM, AFM and XRD. Figure 1 presents the experimental results; TEM (Figure 1A) shows that the N-GQDs have a good dispersity, with most N-GQDs being circular nanosheets; the statistical calculation for more than 100 N-GQDs indicated that the average diameter of the N-GQDs was about 2.65 nm (Figure 1B); the clear and regular lattice fringes in the HRTEM image (Figure 1C) indicate that the interplanar spacing of the N-GQDs was 0.213 nm, which is equivalent to graphite carbon [36]; AFM images (Figure 1D,E) indicate that the heights of the N-GQDs were in the range of 1.5–4.5 nm, implying that the N-GQDs contained 4–12 layers of graphene [37]; the XRD pattern of the N-GQDs indicates a wide diffraction peak at 2θ = 21.06°, corresponding to the (002) crystal facet of graphene [38]. According to the Bragg equation 2dSinθ = λ [39], the layer spacing was about d = 0.36 nm, which is larger than that of graphite (0.335 nm) [40]; the main reasons for this are the nitrogen doping of the GQDs and the oxygen-containing functional groups at the edges of the GQDs [41].

### 3.2. Surface Chemistry of the N-GQDs

**Figure 2:**
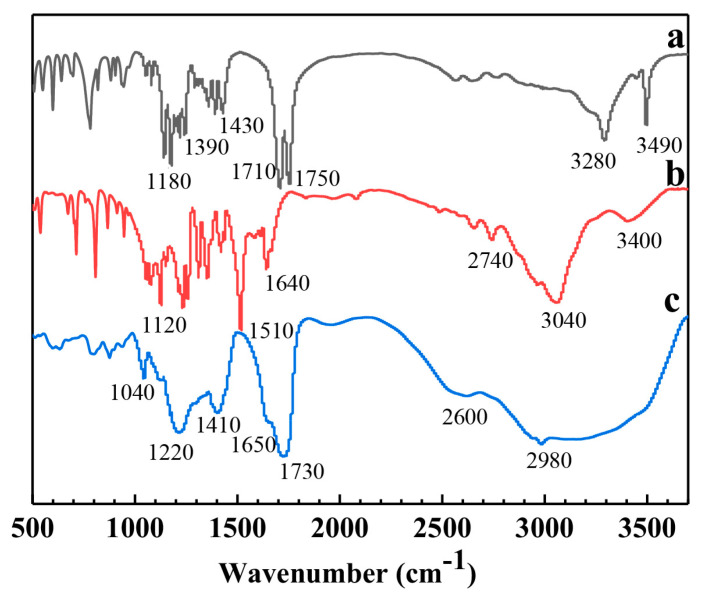
FTIR spectra of (**a**) CA, (**b**) L-Glutamic, and (**c**) N-GQDs.

**Figure 3:**
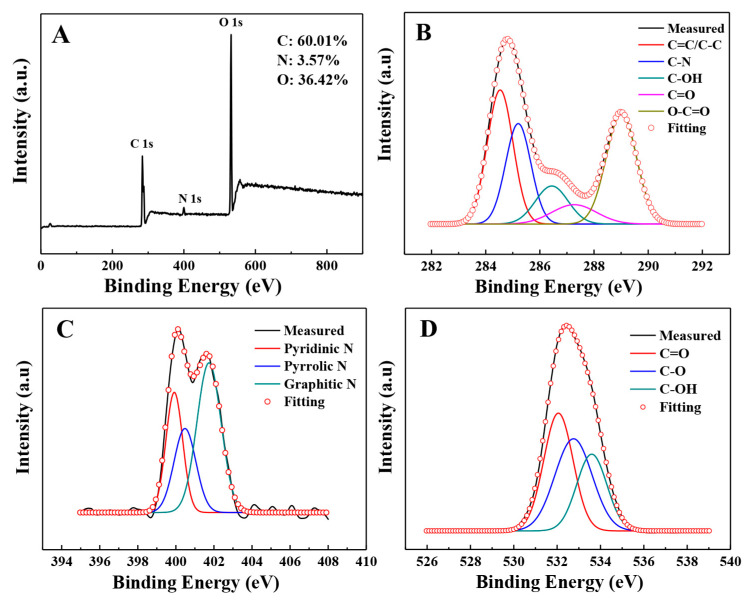
(**A**) Full scan XPS spectrum of the N-GQDs, and high solution XPS spectra of (**B**) C1s, (**C**) N1s, and (**D**) O1s.

The surface chemical architectures of the N-GQDs were detected using FTIR (Figure 2) and XPS (Figure 3). For comparison, the FTIR spectra of pure CA and L-Glu were also measured (Figure 2a,b). The FTIR spectrum of CA shows O–H stretching (3490–3280 cm^−1^), C=O stretching (1750 and 1710 cm^−1^), –CH_2_– scissor vibration (1430 cm^−1^), –CH_2_– oscillation out of plane (1390 cm^−1^), in-plane deformation vibration of C–OH bond (1180–1290 cm^−1^ and 1310–1360 cm^−1^), twisting vibration of –CH_2_– (1220–1240 cm^−1^), C–O stretching (1080–1140 cm^−1^) and C–C stretching (1050 cm^−1^) peaks (Figure 2a) [42,43]. The FTIR of L-Glu (Figure 2b) exhibits O–H and N–H stretching (2780–3600 cm^−1^), –C–H stretching (2650–2740 cm^−1^), carboxylic acid of C=O stretching (1740 and 1640 cm^−1^), N–H out-of-plane bending vibration (1510 cm^−1^), C–N stretching (1420 cm^−1^), –CH_2_– oscillation out of plane (1360 cm^−1^), twisting vibration of –CH_2_– (1250–1310 cm^−1^), in-plane deformation vibration of C–OH bond (1130 cm^−1^ and 1230 cm^−1^), C–O stretching (1080–1130 cm^−1^) and C–C stretching (1050 cm^−1^) peaks [44]. By contrast, the FTIR of N-GQDs shows the intensification of O–H and N–H stretching, C–O stretching, deformation vibration of C–OH bond and C=O stretching, demonstrating that the oxygen content in the N-GQDs was increased, and that the number of oxygen-containing functional groups was also increased. Notably, the formation of C=C stretching (1650 cm^−1^) and the intensification of −C−H stretching and C–C stretching peaks in N-GQDs, indicating that the formation of sp^2^ (graphitic) or sp^3^ (amorphous) carbon structures [45]. In addition, the distinct characteristic peak at 1220 cm^−1^ belongs to the stretching vibration of the C–O–C bond [46], it was presumably formed by decarboxylation of the CA as conjugating to GQDs. The presence of the N–H bond and the C–N bond on the N-GQDs also show L-Glu conjugated to the GQDs. Our fabrication method demonstrates that GQDs can be amino-functionalized during the synthesis process. Comparison to the two-step approach (first fabrication of GQDs, then amino-modification), the one-step method is easier and saves time. The amino-functionalized GQDs benefit from their biocompatibility; the groups of hydroxyl, carboxyl, carbonyl, and amino in the N-GQDs indicate its good hydrophilicity and stability in solution [47].

The XPS characterization was carried out in order to further analyze the elemental compositions and chemical bonds of the obtained N-GQDs. As illustrated in Figure 3A, the full scan spectrum of XPS exhibits three obvious peaks at 545.0, 402.2 and 298.1 eV, revealing the presence of O1s, N1s and C1s, respectively. Quantitative determination of the XPS spectrum shows that the as-prepared N-GQDs consisted of 36.42% O, 3.57% N and 60.01% C, indicating the successful doping of nitrogen into the GQDs. The C to O atomic ratio for the N-GQDs was about 1.65, which is slightly higher than that reported in [28] (1.51), demonstrating that the N-GQDs have a low oxidation level [48]. The N to C atomic ratio was calculated to be 0.06, which is as same as that reported in [28], but remarkably higher than that of the N-GQDs prepared using the electrochemical method (0.04) [49,50]. The deconvolution of the high-resolution XPS spectra of C1s (Figure 3B) reveals five main peaks, confirming the presence of C=C/C–C (284.5 eV), C–N (285.2 eV), C–OH (286.4 eV), C=O (287.3 eV), and O–C=O (288.9 eV) bonds [51], indicating that the as-prepared N-GQDs are rich in hydrophilic groups, such as hydroxyl, carboxyl, and carbonyl groups on the surfaces of N-GQDs, which is consistent with the result of FTIR. The deconvolution of the N1s spectrum (Figure 3C) shows three peaks at 399.9, 400.5, and 401.7 eV. The fitting results coincide well with the experimental results; the three peaks are attributed to the pyridinic N, pyrrolic N and graphitic N respectively [52], confirming again that nitrogen was partly doped into the GQDs. The fitted peaks at 532.1, 532.8 and 533.6 eV of O1s XPS spectrum shown in Figure 3D can be assigned to the three components C=O, C–O, and C–OH [53], respectively, which is in agreement with the XPS spectrum of C1s.

### 3.3. Optical Properties

**Figure 4:**
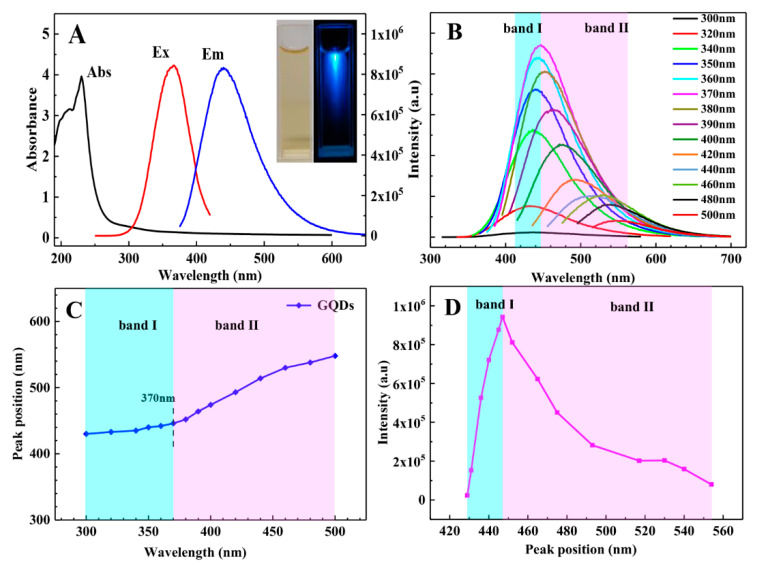
(**A**) The absorption, excitation and emission spectra of N-GQDs. Inset: from left to right are the N-GQDs aqueous solutions under ambient light and UV excitation (irradiated from an optical fiber), respectively; (**B**) the PL spectra of N-GQDs under excited different excitation wavelengths; (**C**) the peak positions and (**D**) the maximum intensities of the PL spectra varying with the excitation wavelengths in (**B**).

The UV-Vis absorption spectra (Figure 4A, Abs) of the N-GQDs aqueous solution shows an obvious absorption peak at 230 nm, which can be attributed to the π→π* electronic transition of the aromatic sp^2^ domains [54]; the absorption peak near 300 nm from the n→π* transition of the heteroatom double bond in GQDs is very weak, indicating the existence of functional groups containing lone pairs of electrons (such as carboxyl groups, etc.) [55]; the excitation (Figure 4A, Ex) and emission (Figure 4A, Em) spectra exhibit good mirror symmetry; the N-GQDs emitted a very bright blue fluorescence that could be observed by naked eye (Figure 4A, inset). When the excitation wavelengths were changed from 300 nm to 500 nm (Figure 4B), the emission peaks of N-CQDs shifted from 430 nm to 548 nm (Figure 4C,D), and the maximum intensity was obtained at the excitation of 370 nm (Figure 4B); when the excitation was in the range of 300–370 nm, the emission peaks were almost invariable (~447 nm), indicating that the emission spectra are excitation independent; thus, from the emission was mainly produced by the carbon core (sp^2^ domain) of the N-GQDs (band I) [56]. Meanwhile, with excitation in the range from 380 to 500 nm, the emission peaks increased with the excitation wavelength, implying that the emission originated from the transitions related to the surface states (hybridization of the carbon backbone and connected chemical groups) (band II). In [55], excitation-dependent emissions were associated with both the n−π* transition of the N/O surface groups and the π–π* charge transfer between the carbon core and the edge of the GQDs. The full width at half maximum (FWHM) of the strongest emission spectrum was only 87 nm. Compared with the reported GQDs synthesized with CA [57], our N-GQDs indicate a narrow emission range; the main reason for this is that L-Glu-functionalized GQDs decrease the surface defects. The QY of the N-GQDs was determined to be 54% (calculation data is shown in Table S1 of Supporting Information). This relatively high QY might be due to the low oxygen content [58], N doping and the protection of the L-Glu as the surface passivation agent. The high QY and narrow FWHM imply that the N-GQDs could be used for imaging or as fluorescent sensors.

**Figure 5:**
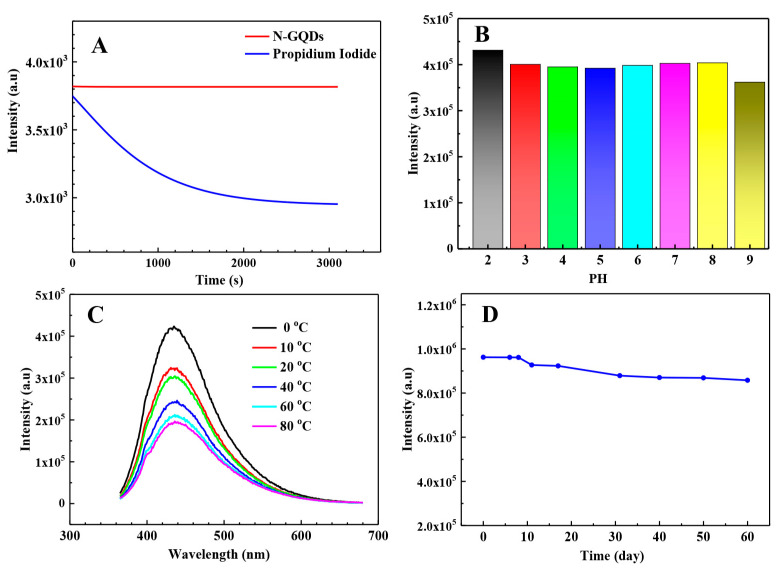
The photo-stability of the N-GQDs. (**A**) The photo-bleaching resistance of the N-GQDs and the traditional dye of propidium iodide; the PL peak intensities varying with (**B**) pH and (**C**) temperature; and (**D**) the PL peak intensities over 2 months.

The photo-stability of the N-GQDs is easily influenced by photo-bleaching, pH value, temperature and preservation time. For practical applications, the photo-stability is very important. Here we probed this question in detail. The results show that the N-GQDs exhibited excellent photo-stability. Under continuous excitation (at 370 nm) for 50 min, the fluorescence intensity remained invariable, indicating the N-GQDs have superior photo-bleaching resistance (Figure 5A). Compared with previously reported N-GQDs [47,59], our N-GQDs show excellent photo-bleaching resistance. For comparison, the traditional organic dye Propidium iodide (PI) was also continuously irradiated for 50 min (Figure 5A, blue line); after irradiation for 10 min and 50 min, the intensity of PI decreased by 19% and 25%, respectively. The above results show that the prepared N-GQDs not only exhibit high QY, but also excellent photo-bleaching resistance. It can be inferred that the N-GQDs should be an excellent candidate for bioimaging.

The fluorescence intensities of the N-GQDs varying with pH value in the range of 2–9 (adjusted by HCl and NaOH) are shown in Figure 5B. Because the intensities did not show any obvious change with pH values in the range of 3–8, it was concluded that the as-prepared N-GQDs exhibit good stability under acid and neutral pH environments, and thus the protonation or deprotonation of the N-GQDs is also very weak [60,61,62]. Compared with the results reported in [63], the as-prepared N-GQDs can be used in a wide range of pH environments. Thus, the N-GQDs have good prospects for the fluorescence imaging of cells and other living organisms.

The thermal stability of N-GQDS is also an important parameter for their application. The emission spectra of the N-GQDs were measured in the range of 0–80 °C. As shown in Figure 5C, the peak positions of the emission spectra were close to 447 nm, but the intensities decrease with increasing temperature, indicating that the thermal stability of the N-GQDS is not so good; the peak intensity at 40 °C is 80% of that at 20 °C.

Whether the N-GQDs can be preserved for a long time is also a significant index for their application. As shown in Figure 5D, the emission spectra of the N-GQDs were measured over a 60 d storage period. After storage at 4 °C for 8, 11, 17, 31, 40 and 60 days, the peak intensities at 447 nm of the emission spectra were 99.66%, 96.13%, 95.68%, 91.10%, 90.20% and 88.92% of the as-prepared N-GQDs, indicating that the N-GQDs can be stably preserved for at least 40 days.

The above results show that the ultrasonic hydrothermal approach is facile for obtaining N-doped GQDs with excellent photo-physical properties and stability.

### 3.4. Mechanism of Photoluminescence

To further investigate the emission mechanism of the N-GQDs, the fluorescence lifetime of the N-GQDs was measured using the time-correlated single photon counting (TCSPC) technique. The fluorescence lifetime of the N-GQDs was detected using an Edinburgh F900 time-resolved fluorescence spectrometer with an LED (370 nm). The monitor emission wavelength of the N-GQDs is 447 nm. The decay curve could be well fitted as a three-exponential function, and contains two fast decays (0.62 ns and 2.38 ns) and one slow decay (16.65 ns), which implies that the N-GQDs have two emission centers; the slow component lifetime is suggested to be related to the surface states in N-GQDs, while the quick lifetimes are related to the carbon core of the graphene structure in the N-GQDs [64]. The decay lifetimes are in good agreement with carbon-based quantum dots grown using different methods, such as chemical exfoliation [65] and the electrochemical method [66]. The average exciton lifetime (*τ_av_*) is 2.653 ns. Additionally, the radiative (*κ_r_*) and nonradiative decay rate constants (*κ_nr_*) can be obtained on the basis of the measured QY (*φ*) and average PL lifetime (*τ_av_*) using the following equations [67]:

(1) $$\kappa_{r}=\Phi /\tau_{av}\cdot$$

(2) $$\kappa_{r}+\kappa_{nr}=1/\tau_{av}$$

The results are *κ*_r_ = 2.04 × 10^8^ s^−1^ and *κ_nr_* = 1.73 × 10^8^ s^−1^.

**Figure 6:**
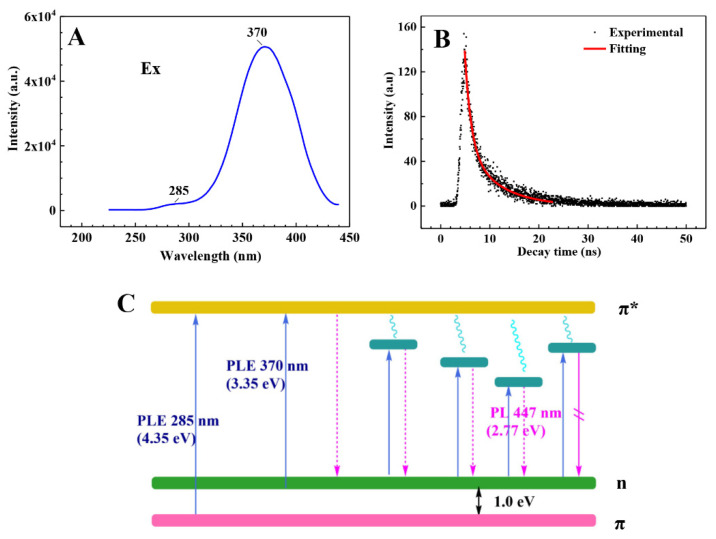
The photoluminescence mechanism of the N-GQDs: (**A**) the excitation spectrum obtained under the monitor emission wavelength at 447 nm and the excitation in the range of 200–430 nm; (**B**) the fluorescence lifetime of the N-GQDs; and (**C**) the schematic energy level of the N-GQDs.

Since both the source (CA and L-Glu) and the solvent (DI water) exhibit extremely weak UV absorption and emission, there should be a fluorescence emission originating from the N-GQDs. The fluorescence emission of the L-Glu-passivated N-GQDs can be attributed to the π electron transition of C=C in the core of N-GQD, which consists of a graphene structure and the surface groups of the N-GQDs [68]. As demonstrated above, our N-GQDs consist of a carbon core, as well as O-, H-, and N-containing functional groups on the surfaces of the N-GQDs. On the basis of the FTIR and XPS results, it can be seen that there are different kinds of functional groups (C-OH, C=O, C-O-C, C-H, C-N, and N-H) present on the surfaces of the N-GQDs; “surface states” are formed via the hybridization of the carbon backbone and the connected chemical groups, and the corresponding energy levels are situated between the π and π* states of sp^2^ C [69]; therefore, the distributed surface states are a reasonable explanation for the difference in chemical bonding in the GQDs. The absorption and emission transitions of the N-GQDs and their energy levels are shown schematically in Figure 6C. In Figure 4C, the excitation-independent emission corresponds to excitation wavelengths of less than 370 nm; therefore, the energy difference between π and n can be estimated on the basis of the intrinsic excitation at 285 nm (4.35 eV) and 370 nm (3.35 eV) (Figure 6A), which is about 1.0 eV. The PL spectra from the carbon core of the graphene structure in the GQDs does not vary with excitation wavelength [56]; therefore, band I (in Figure 4B–D) is excitation independent. The surface states have various energy levels [70]; when a certain excitation wavelength illuminates the N-GQDs, a surface state emission dominates the emission; as the excitation wavelength changes, another corresponding surface state emission may become dominant. In addition to this, the electrons excited to π* may relax into surface states, emitting via radiative combination or not emitting via nonradiative combination. Thus, the excitation-dependent PL of the GQDs (band II in Figure 4B–D) is mainly a result of the surface states.

### 3.5. Fluorescence Cell Imaging with the N-GQDs

**Figure 7:**
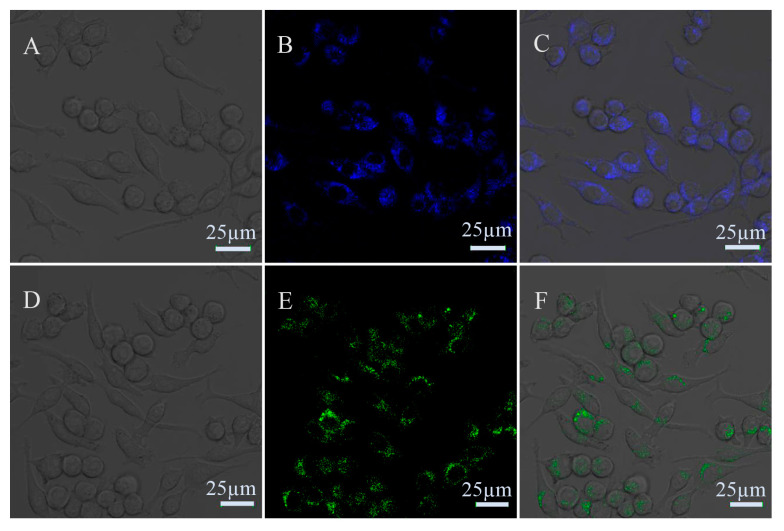
Laser scanning confocal fluorescence microscopy images of BV-2 cells: (**A**,**D**) the cells imaged under bright field, (**B**) 405 nm laser excitation, (**C**) overlay of (**A**,**B**), (**E**) 488 nm laser excitation, and (**F**) overlay of (**D**,**E**).

Most GQDs can be used for biomedical imaging because of their low cytotoxicity, excellent biocompatibility, high fluorescent QY, and excellent photo-bleaching resistance [71,72]. Herein, we used N-GQDs as fluorescent probes for the imaging of BV2 cells. Briefly, BV2 cells were placed on the confocal plate. The cells were cultured in Dulbecco’s modified Eagle medium (DMEM) containing 1% penicillin–streptomycin and 10% fetal bovine serum (FBS) in an incubator with 5% CO_2_ and 95% humidity at 37 °C. The culture solution was changed every other day. When the cell density reached about 80% (~5 × 10^4^ cells/mL), 200 μg/mL N-GQDS was added to the cell medium and cultured at 37 °C and 5% CO_2_ for 1 h. Finally, the BV2 cells were washed three times using PBS buffer (pH 7.4), and the morphology of the BV2 cells was observed and imaged using confocal LSM. The cells displayed enhanced blue (405 nm laser excitation) or green (488 nm laser excitation) fluorescence around their nucleus (Figure 7), indicating that the N-GQDs were able to label the cell membrane and the cytoplasm. Studies have shown that N-GQDs are likely to enter the cytoplasm, which can be attributed to the smaller amount of carboxyl on the surface of N-GQDs [68,73,74,75,76]. The abundant surface functional groups in N-GQDs (carboxyl, carbonyl, hydroxyl, and amino) ensure that they adhere easily to the negatively charged cell membrane [77,78,79], thus achieving effective uptake by cells. By comparing the bright field with the dark field images, the number of stained cells accounted for more than 90%, demonstrating the low cytotoxicity and good biocompatibility of the N-GQDs.

## 4. Conclusions

The ultrasonic-assisted hydrothermal method is a facile method for obtaining bright blue fluorescent N-doped GQDs with CA as a precursor and L-Glu for N doping. The morphology, size, structure, surface chemistry, optical properties, and stability subject to photo-bleaching, temperature, pH and preservation period of the N-GQDs were investigated in detail. The results showed that the N-GQDs possess sizes in the range of 3–6 nm, with an average size of 2.65 nm, containing 36.42% O, 3.57% N and 60.01% O, and possessing good water solubility, outstanding optical properties, excellent photo-bleaching resistance and stability, and good biocompatibility. The PL spectra of the N-GQDs are excitation dependent with excitation in the range of 300–370 nm and excitation independent with excitation in the range of 380–500 nm for core and surface state emissions. The QY reaches up to 54%. At room temperature and in the pH range of 3–8, the fluorescence of the N-GQDs is almost invariable. The N-GQDs can be stably preserved for at least 40 days. The average decay lifetime of the N-GQDs was measured to be 2.653 ns. The calculated radiative and nonradiative decay rate constants were 2.04 × 10^8^ s^−1^ and 1.73 × 10^8^ s^−1^, respectively. The PL mechanism was explained qualitatively. The N-GQDs were used for BV2 cell imaging and showed good results, implying great potential applications for bioimaging or biomarking.

## Data Availability

The data used to support the findings of this study are available from the corresponding author upon request.

## Supplementary Materials

The following are available online at https://www.mdpi.com/article/10.3390/nano11112798/s1, Figure S1: The absorption and PL spectra of N-GQDs obtained under different conditions, Table S1: Quantum yields of N-GQDs under different reaction conditions.
