# Supplementary material for: One-Pot Synthesis of Bright Blue Luminescent N-Doped GQDs: Optical Properties and Cell Imaging
Source: Nanomaterials (Basel). 2021 Oct 22;11(11):2798. doi: 10.3390/nano11112798 (PMC8623353; doi:10.3390/nano11112798)
Supplement: Supplementary file 1 [file nanomaterials-11-02798-s001.zip › nanomaterials-1415701-supplementary.pdf]

Supporting information

One–Pot Synthesis of Bright Blue Luminescent N-Doped GQDs: Optical Properties and Cell Imaging

Huaidong Wang [1], Chong Qi [1], Ailing Yang [1,*] Xiaoxu Wang [2], and Jie Xu [2]

[1] College of Physics & Optoelectronic Engineering, Ocean University of China, Qingdao 266100, China; wanghuaidong@stu.ouc.edu.cn （H.Wang）; qichong@stu.ouc.edu.cn (C. Qi)

[2] College of Food Science & Engineering, Ocean University of China, Qingdao 266003, China; wangxx0416@163.com (X. Wang), xujie9@ouc.edu.cn (J. Xu)

* Correspondence: Correspondence: ailingy@ouc.edu.cn (A. Yang)

1. *Detection of fluorescence quantum yields*

Rhodamine B was as a standard fluorescent reference substance. The UV-Vis absorption spectra of the standard and the N-GQDs were measured (the absorbance must be less than 0.05 at 350 nm). Under excitation at 350 nm, the fluorescent spectra of N-GQDs and rhodamine B were obtained. The QY was calculated by the following formula,

$$Y_u = Y_s(F_u/F_s)(A_s/A_u) \tag{1}$$

in which, the subscript u and s stand for the sample and the reference substance; *F* is the integral area of the fluorescence emission spectrum in the range of 365-680 nm; and *A* is the absorbance.

2. *Synthesis of N-GQDs*

The reaction temperature, time, mass ratio between CA and Glu, and the concentration of reaction solution have significant effects on the formation of N-GQDs. We probed the influences from the four factors on the optical properties of the produces. In each set of experiment, one of parameters of temperature, time, mass ratio between CA and L-Glu, and concentration was changed and kept other three parameters as constants. Step by step, the optimized experimental condition was determined. Figure S1 shows the absorption spectra and PL spectra of the N-GQDs under four different conditions, and Table S1 indicates the corresponding quantum yields (QYs). From Figure S1A and Table S1, the strongest PL was obtained at 180 ℃ as changing temperature, the best QY 46.39 %, thus 180 ℃ was as optimized reaction temperature. Similar to above experimental process, we could obtain the optimized reactive time, mass ratio between CA and Glu, and concentration are respectively 3 h, 1:0.5, and 1.8 g/30 mL. Assumed optimized condition, the QY of N-GQDs is up to 54%.

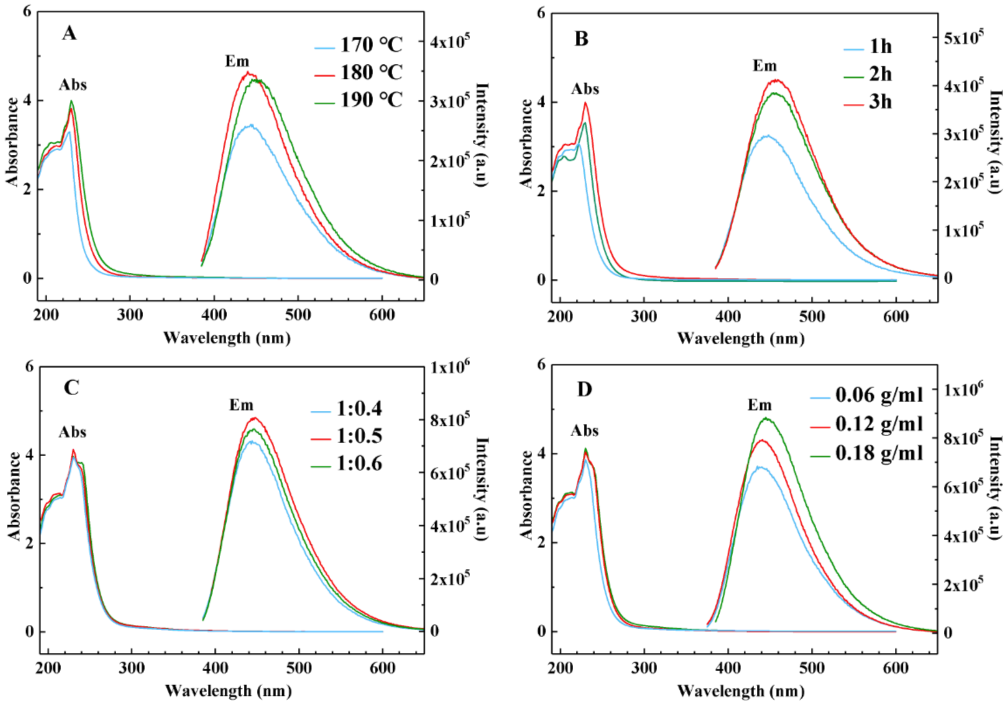

Figure S1. The absorption and PL spectra of N-GQDs obtained under different conditions, in each set of experiment, one of parameters of temperature (**A**), time (B), mass ratio between CA and L-Glu (C), and (D) concentration was changed and kept other three parameters as constants.

Table S1. Quantum yields of N-GQDs under different reaction conditions.

Temperature (°C)	Fu	Au	Fs	As	Yu
170	5976425	0.040	16710320	0.039	22.66%
180	11926485	0.039	16710320	0.039	46.39%
190	10436275	0.039	16710320	0.039	40.60%

Time (h)	Fu	Au	Fs	As	Yu
1	6424315	0.041	16710320	0.039	23.77%
2	10960555	0.039	16710320	0.039	42.63%
3	11926485	0.039	16710320	0.039	46.39%

Mass ratio	Fu	Au	Fs	As	Yu
1:0.4	12495915	0.041	16710320	0.039	46.24%
1:0.5	15664955	0.044	16710320	0.039	54%
1:0.6	10603130	0.040	16710320	0.039	40.21%

Concentration (g/30 mL)	Fu	Au	Fs	As	Yu
0.6	11926485	0.039	16710320	0.039	46.39%
1.2	13310660	0.042	16710320	0.039	48.08%
1.8	15664955	0.044	16710320	0.039	54%
